# Diagnostic sign of intra uterine extra amniotic adhesions with 4D ultrasonography: Sheet on string

**DOI:** 10.4274/tjod.22309

**Published:** 2018-06-21

**Authors:** Eray Çalışkan, Rukiye Ada Bender, Reyhan Aslancan

**Affiliations:** 1 Bahçeşehir University Faculty of Medicine, Department of Obstetrics and Gynecology, İstanbul, Turkey; 2 İstinye University Faculty of Health Sciences, İstanbul, Turkey; 3 Bahçeşehir University Faculty of Medicine, İstanbul, Turkey

**Keywords:** Extra amniotic adhesions, 4D ultrasonography, sheet on string

## Dear editor;

**Figure 1:**
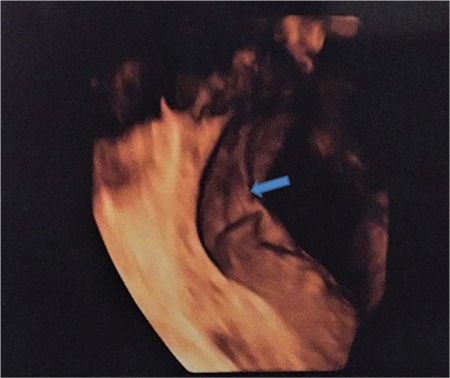
Intrauterine extra-amniotic adhesion demonstrated using 4D ultrasonography; “sheet on a string” appearance

**Figure 2:**
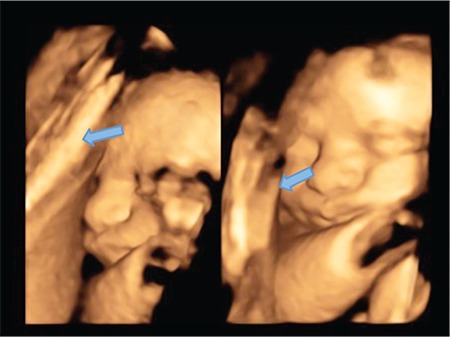
Intrauterine extra-amniotic adhesion demonstrated using 4D ultrasonography; “sheet on a string” appearance

Intra uterine adhesions seen in pregnancy, which were defined in 1894 by Joseph G. Asherman, are divided into two groups as intra amniotic and extra amniotic adhesions. Intra uterine intra amniotic adhesions, which are also known as intra amniotic bands, are easily detectable in first and second trimester ultrasonographic examinations; therefore, close follow-up could provide an appropriate approach. Also intrauterine extra amniotic adhesions are a causative factor of infertility, early pregnancy loss, preterm delivery, cesarean section due to malpresentation, placental invasion abnormalities, and intra uterine fetal death^(1,2)^. There are no ultrasonographic diagnostic signs for intrauterine extra-amniotic adhesions in the literature, and the presumption of negative pregnancy outcomes and following up these patients is difficult for obstetricians. The aim of this study was to provide a handy method to distinguish intrauterine extra- amniotic adhesions for pregnancy outcomes and postpartum follow-up. Based on second trimester detailed ultrasonography outcomes, twenty-four patients were identified as having intrauterine extra amniotic adhesions through 4D ultrasonographic investigations. Sixteen patients had cesarean deliveries; the indications were previous cesarean section for ten, presentation abnormality for four, and placentation abnormality for two of the pregnant women. The intrauterine extra amniotic adhesions were verified during cesarean operations. Eight of the pregnant women had vaginal deliveries and six months after the delivery, adhesions were verified via hysteroscopic imaging. The locations of the adhesions were observed as following: fifteen of the women who had cesarean section had isthmic adhesions (93.75%), and one had cornual adhesion (6.25%). All eight women who had vaginal deliveries had adhesions in the uterine cornu (100%). In conclusion, intrauterine extra-amniotic adhesions can be identified by a specific ultrasonographic appearance known as “sheet on string,” because it looks like a sheet spread out on a string (Figure 1, 2). Intra uterine adhesions that have not been identified in the preconception period but are important for pregnancy follow-up can be determined using 4D ultrasonography during anomaly screening with the advantage of amniotic fluid’s image quality increasing effect.
